# Supplementary material for: Relative, local and global dimension in complex networks
Source: Nat Commun. 2022 Jun 2;13:3088. doi: 10.1038/s41467-022-30705-w (PMC9163155; doi:10.1038/s41467-022-30705-w)
Supplement: Supplementary file 1 — Supplementary Information [file 41467_2022_30705_MOESM1_ESM.pdf]

Supplementary Information: Relative, local and global dimension in complex networks

Robert L. Peach, Alexis Arnaudon, Mauricio Barahona

May 11, 2022

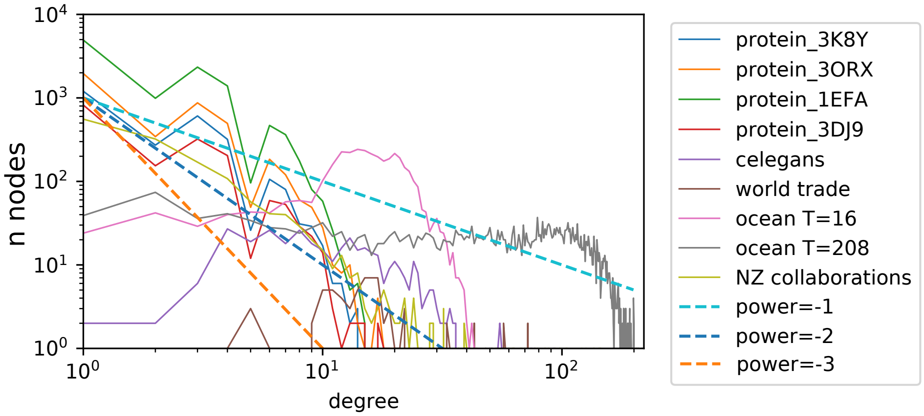

Figure S1: **Supplementary Figure 1** Degree distributions of some of the networks considered in this manuscript. Isolines corresponding to power-law distributions are shown as dashed lines.

1 Degree distributions

We display the degree distributions of the main networks used within this manuscript in Figure S1. Our measure of dimension is applicable to networks of any structure, including those with non-trivial topological features, namely complex networks. We find that the protein networks display a distribution that is resembles a power-law, and thus can be considered to have some 'scale-free' properties. Other networks can be considered more homogeneous in terms of their degree distribution, including the NZ collaboration social network.

2 C. Elegans

Analysis of the undirected connectome (N=377) of the nematode *C. elegans* with the inclusion of muscles, important for examining control [1][1], and where scales have previously been shown as important [2]. We highlight the top 40 local dimension neurons in Table S1.

[1] https://www.wormatlas.org/neuronalwiring.html

Neuron	Neuron type	local_dimension	z-score
AVAL	I	2.563	2.372
AVBL	I	2.554	2.304
AVAR	I	2.545	2.238
AVBR	I	2.543	2.224
AVER	I	2.536	2.171
RICL	I	2.520	2.050
ADAR	I	2.513	1.998
AVDL	I	2.512	1.988
RIGL	I	2.512	1.987
AVEL	I	2.509	1.964
AVDR	I	2.508	1.956
RIS	I	2.504	1.929
RMGL	M	2.494	1.850
RICR	I	2.488	1.807
ADEL	S	2.485	1.787
RMGR	M	2.482	1.759
RIML	M	2.479	1.739
SDQR	I	2.479	1.739
RIMR	M	2.475	1.712
ADER	S	2.475	1.712
PVCL	I	2.474	1.704
AVKL	I	2.470	1.670
PVCR	I	2.470	1.669
ALA	I	2.467	1.650
AVKR	I	2.460	1.595
RIGR	I	2.455	1.561
SMDDR	M	2.451	1.528
SAAVR	I	2.449	1.513
RIAR	I	2.446	1.493
ALML	S	2.441	1.452
PVNR	I	2.431	1.374
SAAVL	I	2.429	1.365
RIBR	I	2.429	1.361
RIAL	I	2.428	1.355
RIBL	I	2.427	1.350
ADAL	I	2.419	1.286
AIZR	I	2.416	1.267
FLPR	S	2.415	1.259
PVPR	I	2.415	1.256
SABD	I	2.413	1.243

Table S1: **Supplementary Table 1** The 40 neurons with the highest local dimension. The z-score is calculated in respect to the mean and standard deviation of the full set of neurons.

Supplementary References

[1] Gang Yan, Petra E Vértes, Emma K Towlson, Yee Lian Chew, Denise S Walker, William R Schafer, and Albert-László Barabási. Network control principles predict neuron function in the caenorhabditis elegans connectome. *Nature*, 550(7677):519–523, 2017.

[2] Karol A. Bacik, Michael T. Schaub, Mariano Beguerisse-Díaz, Yazan N. Billeh, and Mauricio Barahona. Flow-Based Network Analysis of the Caenorhabditis elegans Connectome. *PLoS Comp. Biology*, 12(8), 2016.
